# The effect of microwave stabilization on the properties of whole wheat flour and its further interpretation by molecular docking

**DOI:** 10.1186/s13065-021-00782-x

**Published:** 2021-10-16

**Authors:** Chenling Qu, Qiankui Yang, Lina Ding, Xueke Wang, Shengqiang Liu, Min Wei

**Affiliations:** 1 College of Food Science and Technology, Henan University of Technology, No. 100 of Lianhua Street, Zhengzhou, 450001 People’s Republic of China; 2 School of Pharmaceutical Sciences, Zhengzhou University, Zhengzhou, 450001 People’s Republic of China

**Keywords:** Whole wheat flour, Microwave, Lipase, Properties, Molecular docking

## Abstract

In order to stabilize the whole wheat flour and extend its shelf life, microwave was employed to heat the wheat bran to inactivate the lipase in this paper. The effects of microwave heating of wheat bran on the lipase activities, gluten properties, dough properties and storage stability of the stabilized whole wheat flour, and the quality of steamed bread made of stabilized whole wheat flour were investigated. Furthermore, molecular docking was applied to interpret the mechanism. The results showed that microwave can reduce lipase activity, maintain the quality of whole wheat flour dough and steamed bread, and retard rancidity. The molecular docking results displayed that the conformation of the amino acids chains near the lipase catalytic center changed, which made the substrate difficult to enter the catalytic center and prevented the hydrolysis of the fat substrate.

## Introduction

Whole wheat flour contains all the nutrients from wheat endosperm, germ and bran, including dietary fiber, proteins, vitamins and minerals etc. And food made from whole wheat flour is more popular because it is beneficial to people’s health, e.g., reducing risk of cancer, cardiovascular disease and obesity [1–3].

However, short shelf-life of whole wheat flour limits its use. The quality deterioration of whole wheat flour during storage is mainly due to lipid degradation [4]. Lipid degradation includes lipid hydrolysis and oxidation. Lipid can be hydrolyzed to fatty acids by lipase catalysis. The hydrolysis rate can be measured by the lipase activity [5]. Lipid oxidation includes lipoxygenase catalyzed oxidation and oxidation by oxygen, which can generate volatile compounds with shorter carbon chain, e.g., aldehydes, alkones, esters and furans etc. [6, 7]. These reactions lead to sensory changes and quality deterioration of whole wheat flour.

Lipid degradation is usually accompanied by the changes of gluten function [8], the decrease of the content of vitamin E [9] and the oxidation of carotenoids [10]. Free fatty acids obtained from fat hydrolysis can modify the amylose, which affects the viscosity of amylose [11]. These lipid degradation-induced changes are detrimental to the storage stability of whole wheat flour.

As suggested above, the key to stabilize whole wheat flour and prolong the shelf-life of whole wheat flour is inactivating the enzymes in it. The stabilization methods included heat treatment, autoclave, exposure to infrared, ultraviolet, microwave radiation and γ-radiation etc. to wheat bran [12–15]. Then the treated bran was added to white flour to make stabilized whole wheat flour. Among these methods, microwave was an effective and convenient way to inactive enzymes. It had been reported that microwave radiation could reduce lipase activity greatly and lower increase rate of free fatty acid substantially [11, 16–18].

In this paper, microwave was used to inactivate enzymes in the wheat bran and stabilize the whole wheat flour. Furthermore, the effects of microwave on the quality characteristics of whole wheat flour were investigated. Phospholipase D (PLD), which was widely existed and important in wheat [19], was selected as the template to study its structure changes by molecular docking after heating to explore the stabilization mechanism.

## Materials and methods

### Preparation of stabilized whole wheat flour

Wheat (*Triticum aestivum* L.cv. Zhoumai 27) was obtained from Rui-Xing Seed Corporation (Zhengzhou, China) with initial moisture content 12.7% on the wet basis (w.b.). The wheat kernels were tempered to 14% wet basis (w.b.) and milled by Buhler laboratory mill (MLU-202, Swiss) according to AACC 26-20 (2000) with powder yield 65.7%.

The wheat bran obtained from different mill flows were collected and mixed. Then the moisture content of the mixed wheat bran was adjusted to 20% with distilled water. The mixture was stirred and incubated for 30 min in a sealed plastic bag for moisture equilibrium. After that, the wheat bran with 3 cm thick on a borosilicate glass plate (with diameter 30 cm), was put into a microwave oven (MYE-2070 M, Haier Electronics Group, Qingdao). The wheat bran was heated at 700 W for 60 s, 90 s, 120 s, 150 s and 180 s, respectively. After heating, the temperatures of the wheat bran were monitored by a mercury thermometer (Shuangcheng Thermal Instrumentation, Changzhou, China, 0–150 °C) immediately.

After that, the microwave treated bran was cooled to room temperature and milled by a platform ultra-fine mill (HMB-700S, Hongquan Machinery Cooperation, Taiwan). The stabilized superfine milled wheat bran was added back to the white flour according to the original proportion (powder yield 65.7%) to make stabilized whole wheat flour as samples. The microwave untreated wheat bran was also superfine milled and added back to white flour to make the unstabilized whole wheat flour as the control.

### Determination of lipase (LA) activity

The LA activity of the whole wheat flour was measured by spectrophotometric assay according to Cai et al. [20]. LA was extracted from 1.0 g whole wheat flour by 5 mL Tris–HCl buffer (pH 8.0, 50 mM). The LA extract (200 μL) and 10 mM p-nitrophenyl octanoate (20 μL, as a substrate) were reacted in Tris–HCl buffer (1780 μL). The reaction was performed for 3 min at 37 °C. One unit of LA activity (u) was defined as an increase of 0.1 absorbance at 405 nm within 1 min.

### Determination of the functional qualities of whole wheat flour

#### Gluten and farinograph properties of whole wheat flour

Wet gluten contents and gluten index were measured according to GB/T 14608-2003 (Chinese National Standards). Farinograph characteristic of dough were tested by a Brabender Farinograph (300 g steel bowl) according to Liu et al. [21].

#### Pasting properties of whole wheat flour

Rapid visco analyzer (RVA, Newport Scientific, Sydney, Australia) was used to investigate the pasting properties according to Fierens et al. [22].

#### Steamed bread preparation and its texture tests

1.5 g yeast and 115–125 mL warm distilled water were added to 200 g whole wheat flour. The mixture was stirred for 3 min in a dough mixer (JHMZ200, East Fude Technology Development Center, Beijing) to form dough. The dough was then sheeted eight times to exclude air. Then the dough was cut into three pieces of equal mass. After that, each piece was kneaded to round with smooth surface by hand. The round dough was fermented at 30 °C, 85% RH for 70 min and then the proofed dough was steamed for 20 min.

Texture analyzer (TA-XT Plus, Stable Miero System, Britain) with a P36 probe was used to determine the textures of the steamed bread. After cooling for 1 h, steamed bread was sliced to 20 mm thick by a bread slicer (SM302N, Xingmai Machinery Cooperation, Wuxi City). Pre-test speed, test speed, post-test speed were set as 2.0 mm s^−1^, 1.0 mm s^−1^, 1.0 mm s^−1^, respectively and trigger force as 5 g. Steamed bread piece was compressed to 50% of its height. Interval between two compressions was 5 s and compression time was 2 s. The hardness, adhesiveness, resilience, cohesiveness and springiness of the steamed bread were tested.

### Whole wheat flour storage

Stabilized (microwave treated bran) whole wheat flour (Treated, T) and unstabilized whole wheat flour (Control, C) were packed by polyethylene bags, respectively. Each bag contained 100 g whole wheat flour. The bag was sealed by a heat sealer (SF300, Chuliang Hardware Tool Corporation, Taizhou of Zhejiang province) and then placed at 25 °C in a climate incubator (Yuejin Medical Instrument Corporation, Shanghai City).

Free fatty acid (FFA) values were monitored by titration according to Chinese National Standard GB/T 5510-2011. Lipid was extracted by benzene and titrated with 0.01 M KOH with 95% ethanol as solvent. The phenolphthalein dissolved in 95% ethanol was used as an indicator. FFA value was expressed as the KOH consumption amount in 100 g sample (mg KOH·100 g^−1^).

### Molecular docking

#### Homology modeling of phospholipase D (PLD)

Using PLD amino acid sequence (Uniprot, ID: W5AEP3) as the probe, the homologous template protein was obtained by homology search using the online BLAST module of Uniprot (http://www.uniprot.org/) site. With the sequence alignment comparison with the MOE 2015.10 Alignment module, the Homology Model module was used for homology modeling, and the model was evaluated by Psi-Phi Plot to determine its rationality.

#### Kinetic simulation of PLD

**Fig. 1:**
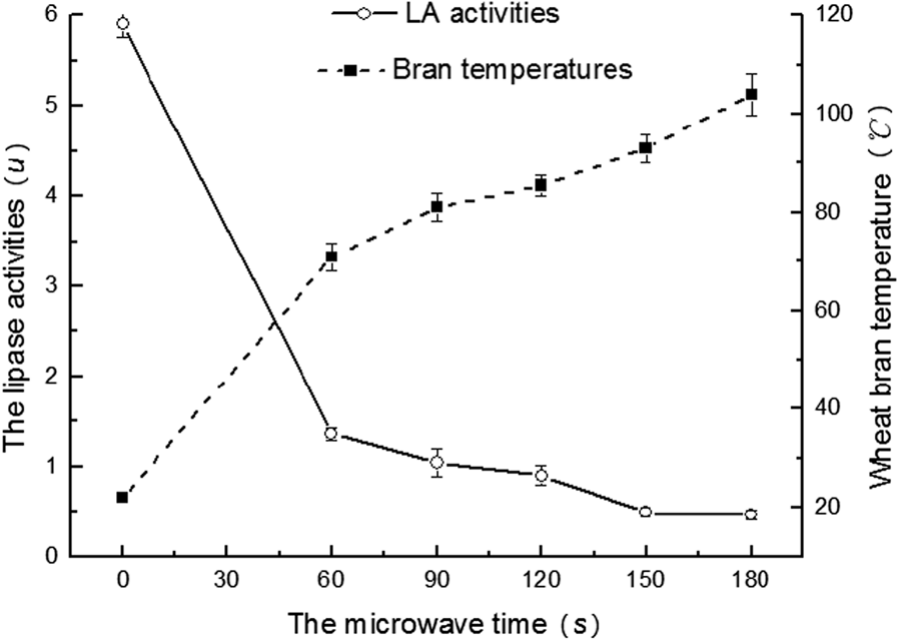
The activities of lipase in stabilized whole wheat flour and the wheat bran temperatures after different microwave heating

In order to further optimize the initial model and investigate the model structure changes of PLD at higher temperature, the 14 ns dynamic simulation was conducted by NAMD 2.11 [23] at 300 K (27 °C, room temperature) and 353 K (80 °C, lipase activity was greatly reduced at this temperature, Fig. 1), respectively. Two simulation systems were constructed, adding 10 Å TIP3 periodic water box and counter ion around the protein. First, the solute was subjected to 5000 step energy minimization, and then 2000 step energy minimization to eliminate the adverse collisions between the residues within the model. After that, two systems were heated twice to the specified temperature and balanced 60 ps under NVT condition. At last, the two system 14 ns dynamic simulation were carried out in the NPT system and the average conformation after stabilization were adopted to study.

### Statistical analysis

Data were analyzed by ANOVA [24, 25], and the Ryan-Einot-Gabriel-Welsch (REGWQ) multiple range test was used for significant difference determination at the *P* < 0.05 level [26] by SPSS software (version 21.0).

## Results and discussion

### LA activity changes of whole wheat flour

Figure 1 showed the changes of lipase activities and bran temperatures as the microwave time increased. It can be seen from Fig. 1 that when the bran was heated for 60 s, its temperature rose to 69 °C and the residual LA activities dropped rapidly from 5.90 to 1.35 u. When the heating time exceeded 90 s, LA activities declined more slowly than that in the first 60 s and the corresponding wheat bran temperature was 80 °C. As the bran was heated for 180 s, the residual LA activity was only about 8% of the control. Therefore, it can be concluded that the LA activities of wheat bran could be efficiently reduced by microwave heating.

### Microwave heating effects on the properties of whole wheat flour

#### Gluten and farinograph properties of whole wheat flour

The data in Table 1 presented the changes of wet gluten content, dry gluten content, gluten water absorption and gluten index for the stabilized whole wheat flour. It can be seen from Table 1 that the wet gluten content and dry gluten content increased slightly with the extension of microwave heating time, but there is no statistically significant difference (*P* > 0.05). The gluten index declined a little with the increased heating time. The results indicated that microwave heating had little effect on the properties of gluten. It is because gluten is mainly present in the endosperm of wheat. The sample treated by microwave was mainly composed of wheat bran, aleurone layer and germ. The reduced glutathione [27] and cysteine in the wheat bran and germ were easier to form disulfide bonds after heating [28–32], which made the gluten easier to form and the gluten content increased slightly.

**Table 1:** Effects of different microwave treatment time on whole wheat flour gluten quality

Heating time (s)	Wet gluten content (%)	Dry gluten content (%)	Gluten water absorption (%)	Gluten index
0 (control)	29.2 ± 0.6^a^	11.9 ± 0.3^a^	144.5 ± 1.5^a^	77 ± 3^a^
60	30.5 ± 0.2^a^	12.0 ± 0.4^a^	154.5 ± 1.4^a^	67 ± 3^b^
90	31.5 ± 2.0^a^	12.8 ± 1.0^a^	148.2 ± 35.1^a^	60 ± 2^bc^
120	32.5 ± 0.7^a^	12.4 ± 0.8^a^	162.9 ± 11.5^a^	59 ± 2^bc^
150	33.2 ± 1.5^a^	12.5 ± 0.7^a^	166.8 ± 27.7^a^	60 ± 2^bc^
180	33.4 ± 0.1^a^	14.0 ± 0.9^a^	139.5 ± 15.7^a^	58 ± 1^c^

Data were expressed as means ± standard deviations. Different superscript letters in the same column show a significant difference (*P* < 0.05).

The rheological parameters of whole wheat flour were also affected by microwave heating (Table 2). Compared with the control, microwave heating wheat bran between 120 and 180 s could extend flour dough development time and stability time, reduce weakening degree as well. Therefore, microwave heating to wheat bran for a certain amount of time could improve the dough characteristic of whole wheat flour a little. The results were consistent with Mosharr’s study [31], in which flour samples with hydrothermal treated bran (55 °C, 24 h) showed longer development time, being more stable and displayed a lower degree of softening.

**Table 2:** Effects of different microwave treatment time on whole wheat flour farinograph properties

Heating time (s)	Dough development (min)	Flour water absorption (%)	Dough stability (min)	Dough weaking (FU)	Farinograph quality number (mm)
0 (Control)	3.71 ± 0.10^b^	71.4 ± 0.2^a^	2.99 ± 0.06^b^	122 ± 7^a^	54 ± 1^c^
60	4.37 ± 0.06^a^	70.0 ± 0.7^b^	3.25 ± 0.01^a^	114 ± 1^ab^	64 ± 1^ab^
90	3.79 ± 0.04^b^	71.8 ± 0.8^a^	3.03 ± 0.01^b^	113 ± 1^ab^	57 ± 1^bc^
120	4.19 ± 0.02^a^	69.7 ± 0.1^b^	3.24 ± 0.00^a^	105 ± 4^b^	62 ± 1^abc^
150	4.30 ± 0.12^a^	72.2 ± 0.2^a^	3.35 ± 0.00^a^	108 ± 2^ab^	59 ± 1^ab^
180	4.34 ± 0.07^a^	72.3 ± 0.4^a^	3.28 ± 0.14^a^	101 ± 2^b^	61 ± 1^a^

Data were expressed as means ± standard deviations. Different superscript letters in the same column show a significant difference (*P* < 0.05).

#### Pasting properties of whole wheat flour

From Table 3, it was noted that compared with the control, the peak viscosity, trough viscosity, final viscosity breakdown value and setback value of the stabilized whole wheat flour were significantly increased as microwave heating time increased. Starch paste viscosity was largely determined by the expansion degree of the starch granules. The swelling starch occupied larger volume and aligned more crowded. So, in gelatinization process, starch granules got closer and generated higher internal friction, which obtained the higher viscosity. In our study, the remaining small amount of starch in the bran mixture partly gelatinized after microwave heating, which caused the viscosity property changes of the stabilized whole wheat flour. Some studies have also supported that heat treatment could change the structures of starch granules and enable the starch partly gelatinized, which made the pasting properties change [30, 33–35].

**Table 3:** Effect of different microwave treatment time on whole wheat flour viscosity properties

Time (s)	Peak viscosity (cP)	Trough viscosity (cP)	Breakdown (cP)	Final viscosity (cP)	Setback (cP)	Peak time (min)	Pasting temp (°C)
Control	2733 ± 35^f^	1975 ± 21^f^	758 ± 10^e^	3055 ± 45^f^	1080 ± 9^e^	6.20 ± 0.07^a^	65.9 ± 0.7^a^
60	2759 ± 28^e^	1998 ± 43^e^	761 ± 14^de^	3085 ± 27^e^	1087 ± 15^de^	6.19 ± 0.11^ab^	65.6 ± 2.3^ab^
90	2785 ± 27^d^	2020 ± 30^d^	764 ± 7^ cd^	3115 ± 34^d^	1095 ± 10^ cd^	6.18 ± 0.08^bc^	65.2 ± 1.1^bc^
120	2810 ± 33^c^	2042 ± 27^c^	767 ± 11^bc^	3145 ± 29^c^	1102 ± 12^bc^	6.17 ± 0.13^c^	64.9 ± 1.5^ cd^
150	2836 ± 37^b^	2066 ± 35^b^	770 ± 8^ab^	3175 ± 40^b^	1110 ± 8^ab^	6.16 ± 0.10^d^	64.5 ± 2.1^de^
180	2861 ± 25^a^	2088 ± 19^a^	773 ± 13^a^	3205 ± 45^a^	1117 ± 13^a^	6.15 ± 0.15^e^	64.1 ± 1.6^e^

Data were expressed as means ± standard deviations. Different superscript letters in the same column show a significant difference (*P* < 0.05).

#### Steamed bread quality and its texture

The steamed bread made from whole wheat flour with microwave treated bran showed no difference from that made from control basically. All the groups of steamed breads made of whole wheat flour (stabilized or non-stabilized) were with smooth surface and uniform internal pores. In addition, there was no obvious difference of all the steamed breads in taste. Texture analysis results were shown in Table 4. Compared with the control, the hardness, adhesiveness, springiness, and chewiness of the steamed bread made from stabilized whole wheat flour with different microwave treating time were not significantly changed. Only the resilience showed a slight change.

**Table 4:** Effect of microwave treatment to wheat bran on the texture properties of the steam bread made by the whole wheat flour

Treatment time (s)	Hardness (g)	Adhesiveness (g∙sec)	Springiness (%)	Chewiness	Resilience (%)
Control	8833 ± 297^a^	355 ± 34^a^	87.9 ± 0.6^a^	5595 ± 64^a^	34.9 ± 0.8^ab^
60	11,352 ± 520^a^	374 ± 59^a^	89.6 ± 0.9^a^	6866 ± 468^a^	32.1 ± 0.1^b^
90	12,422 ± 186^a^	323 ± 7^a^	83.2 ± 0.8^a^	6629 ± 128^a^	26.6 ± 0.7^c^
120	9533 ± 744^a^	231 ± 18^a^	85.5 ± 0.3^a^	5779 ± 237^a^	33.7 ± 0.6^b^
150	10,880 ± 355^a^	256 ± 7^a^	88.2 ± 0.6^a^	6661 ± 644^a^	32.3 ± 0.3^b^
180	9709 ± 720^a^	213 ± 34^a^	84.6 ± 1.0^a^	6104 ± 514^a^	38.3 ± 0.2^a^

Data were expressed as means ± standard deviations. Different superscript letters in the same column show a significant difference (*P* < 0.05).

### Storage of whole wheat flour

**Fig. 2:**
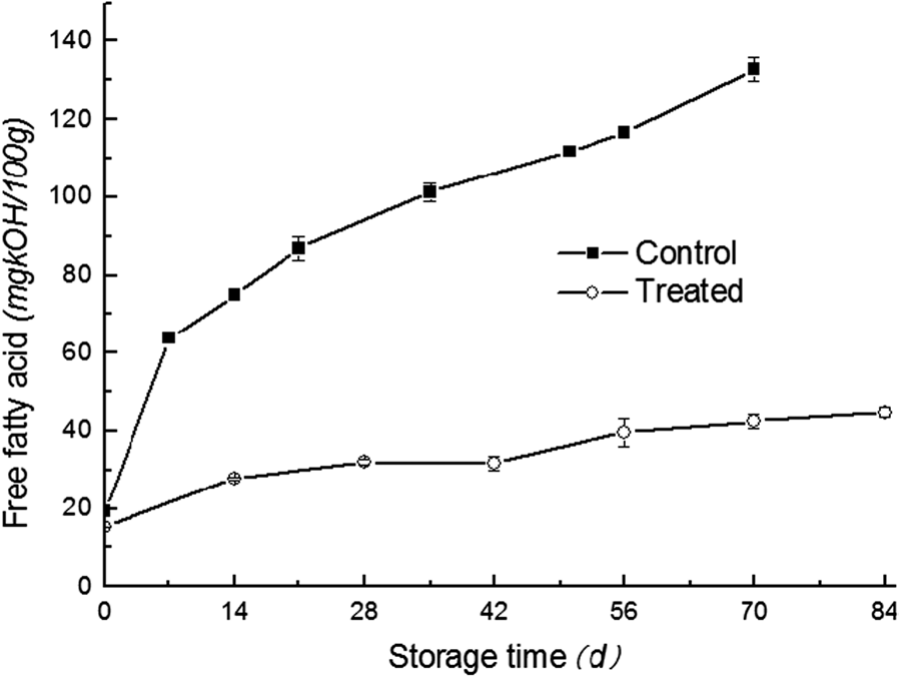
Total free fatty acids accumulated in control and microwave heated whole wheat flour during storage

Microwave stabilized bran (700 W, 180 s microwave treated) added whole wheat flour (Treated, T) and untreated whole wheat flour (Control, C) were stored at 25 °C and the free fatty acid (FFA) values were monitored. As shown in Fig. 2, the FFA values of C increased from 19.3 to 132.7 mg KOH·100 g^−1^ after 70 days’ storage, and that of T increased more slowly from 18.2 to 40.5 mg KOH·100 g^−1^ after 84 days’ storage. Therefore, microwave heating process can definitely slow down the rancidity of whole wheat flour and extend its shelf life.

### Molecular docking

#### Homology modeling of wheat PLD

**Fig. 3:**
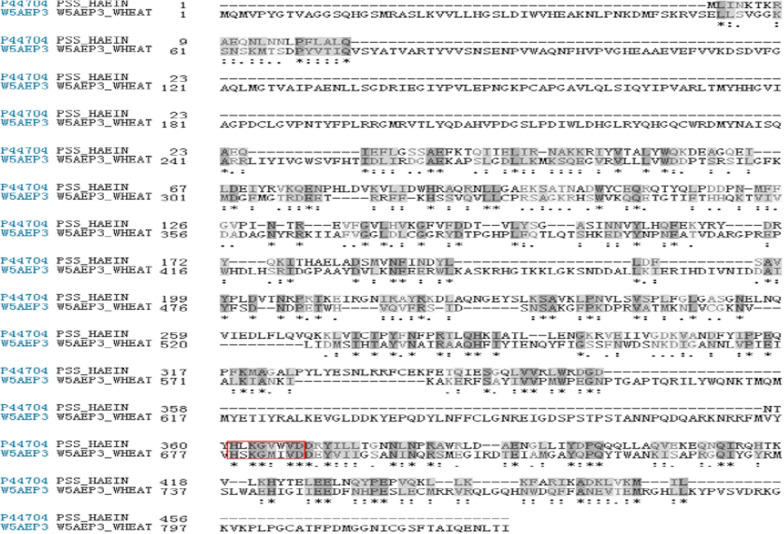
Sequence alignment between wheat PLD and template protein. (The HKD motif was marked in a red box, the template motif which was similar to that of the wheat PLD motif was labeled in gray.)

The amino acid sequence of wheat PLD contains 827 amino acid residues, which had typical C2 domains and two HKD catalytic centers. The HKD active site referred to two H (histidine) K (lysine) D (aspartic acid) motifs, which was a mark motif of PLD and was an active site for catalytic hydrolysis. Because heating led to the LA inactivation, the model construction of active site regions was considered. The CDP-diacylglycerol-serine O-phosphatidyl transferase (PDB ID: 3HSI), which had high homology with the PLD catalytic site of wheat, was selected as the template [36]. Sequence alignment results (Fig. 3) showed that the two contained a same HKD catalytic motif, and the sequence homology near the catalytic site was 28.8%, with a similarity of 48.8%, which basically met the modeling standards.

**Fig. 4:**
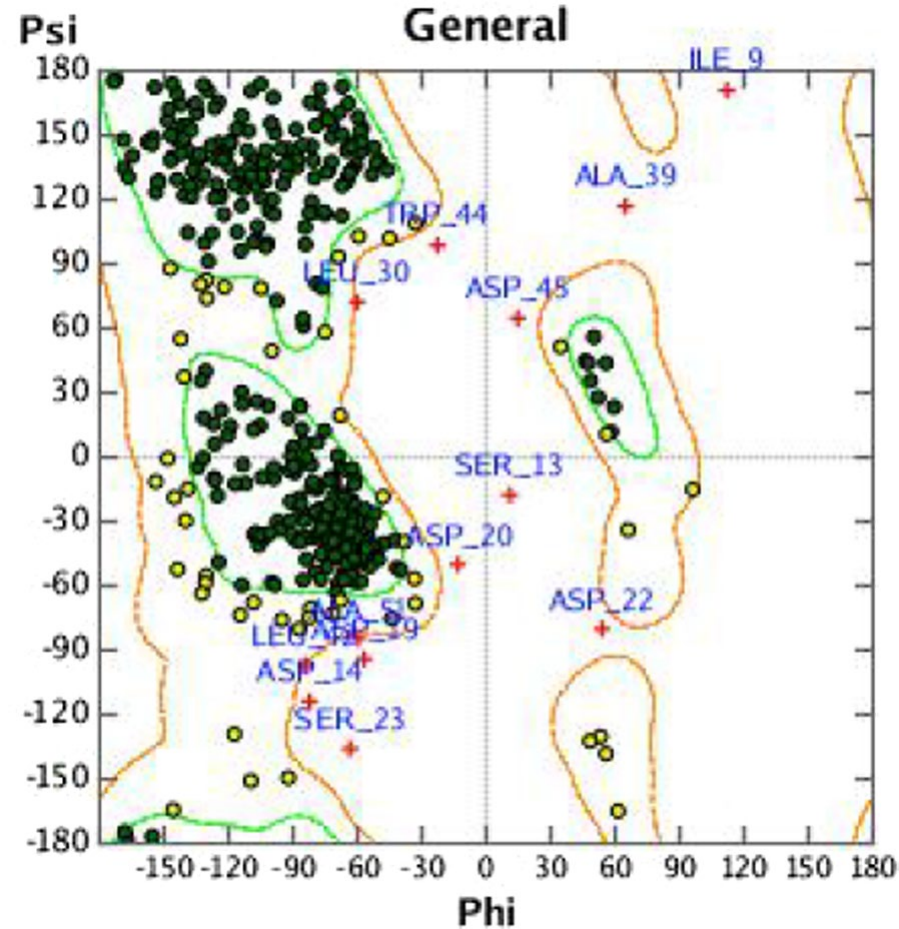
Psi-Phi Plot of wheat PLD model. (The green dots represented the amino acids located in the green core region. The yellow dots represent the amino acids in the yellow acceptable region, and the red cross indicated the amino acids in the unacceptable region.)

Psi-Phi Plot of the obtained wheat PLD structure model displayed that (Fig. 4) 92.9% of the residues was in the best area, 5.6% residues located in the acceptable region, and 1.5% of the residues fell in the disallowed zone, which suggested that the protein model was basically reasonable.

#### Kinetic simulation

**Fig. 5:**
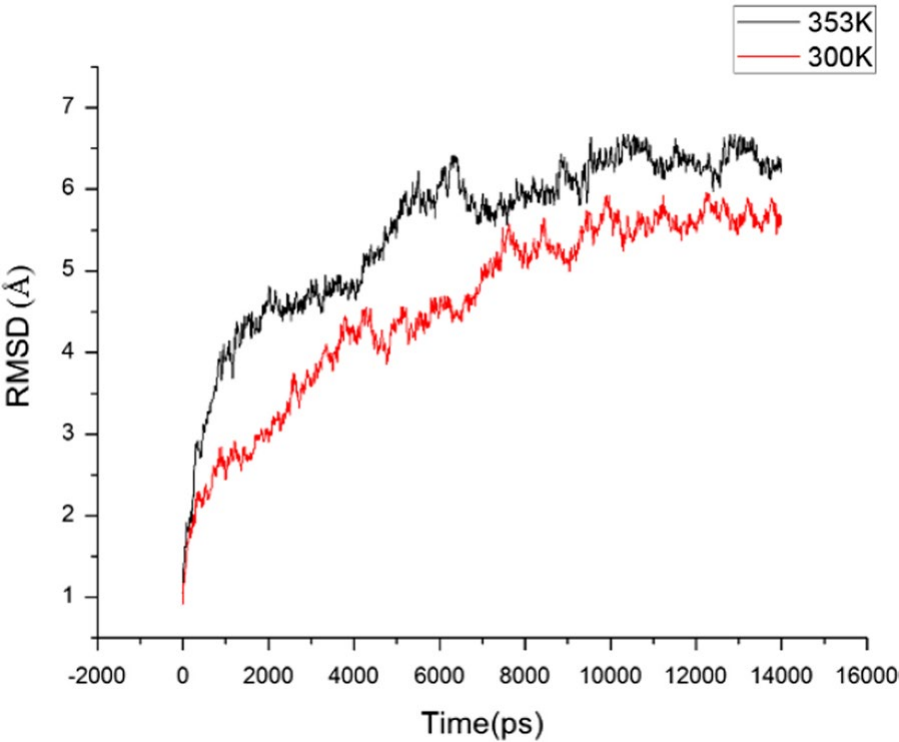
The curve of RMSD versus time in the simulation process

**Fig. 6:**
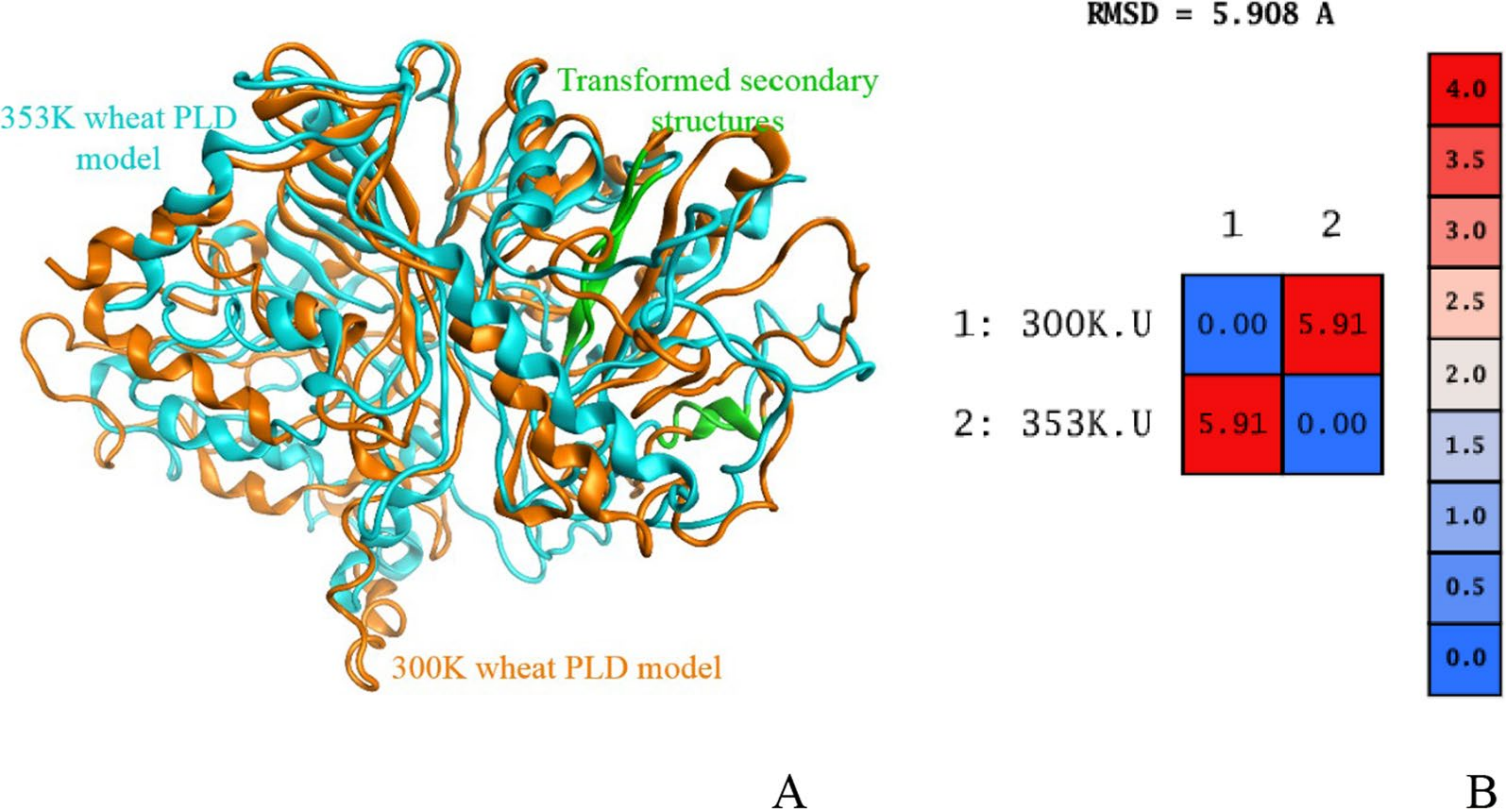
**A** The wheat PLD superposition structure at 300 K (Orange) and 353 K (cyan). **B** RMSD values of the superposition structure

Two wheat PLD protein models at 300 K (27 °C) and 353 K (80 °C) were obtained by kinetic simulation. The curve of Root Mean Square Deviation (RMSD) versus time in the simulation process was exhibited in Fig. 5. It can be seen in Fig. 5 that the two models were basically stable after 10 ns. The RMSD of wheat PLD model at 300 K and 353 K were fluctuated at 5.5 Å and 6.3 Å, respectively after equilibrium. It can be concluded that heating had obvious effect on wheat PLD protein structure. The protein superposition structure at two temperatures (Fig. 6) showed that after heating, the surface of the structure changed more obviously, and the secondary structure also transformed.

**Fig. 7:**
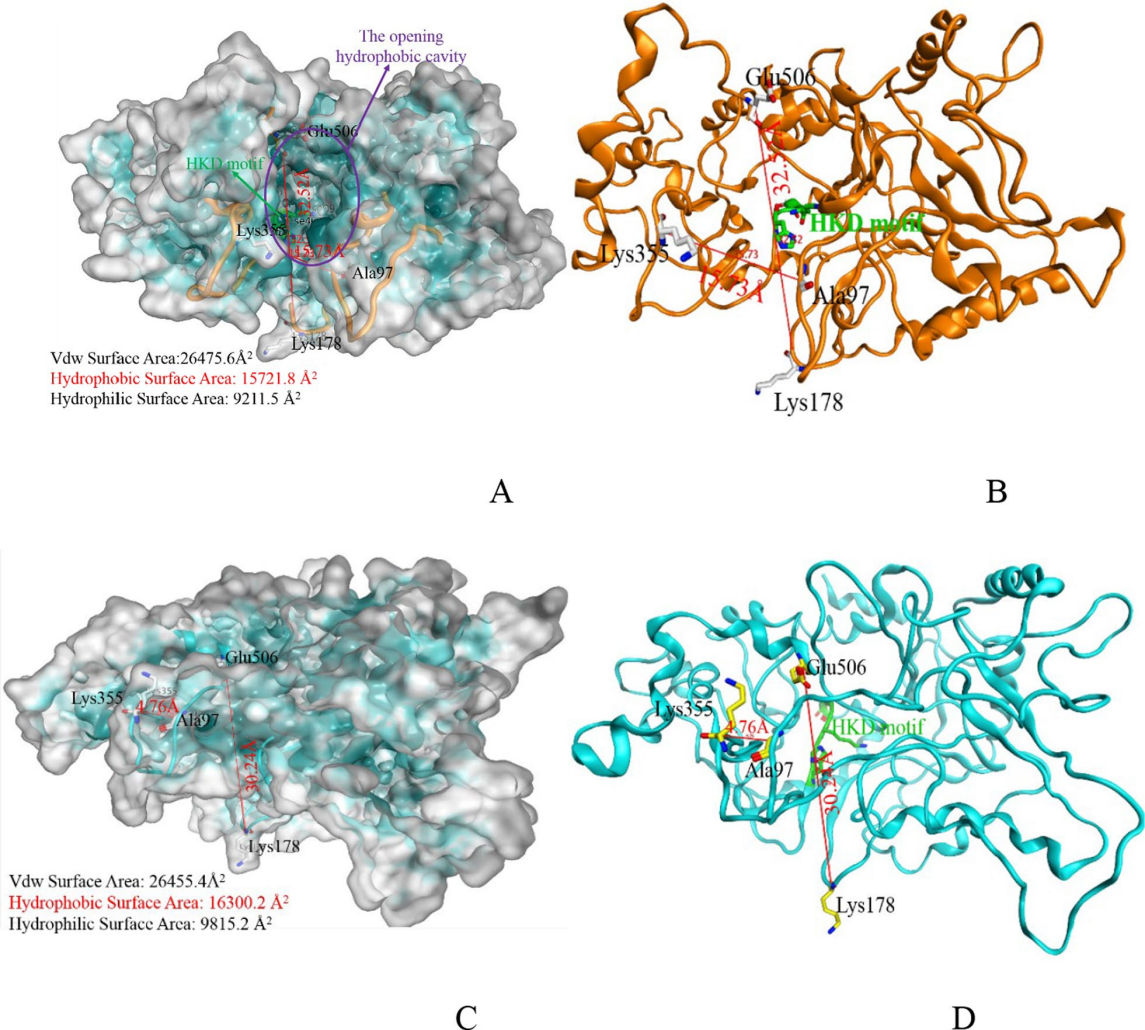
**A**, **B** Wheat PLD structure at 300 K; **C**, **D** Wheat PLD structure at 353 K. (The number in red indicated the distance between the four residues located at the opening of hydrophobic cavity. The green residue was HKD motif, and the hydrophobic surface was displayed in cyan.)

The modeling structure showed that HKD catalytic center was located in an open internal hydrophobic cavity (Fig. 7A and B). The distances between Lys355 and Ala97 α-C at 300 K and 353 K were 15.73 Å and 4.76 Å, respectively. And the distances between Glu506 and Lys178 α-C were 32.52 Å and 30.24 Å, respectively. These changes resulted in the cavity being covered (Fig. 7C and D) and the substrate cannot approach to the catalytic domain. It may be the reason that heating led to inactivation of the lipase. Moreover, protein surface (Fig. 7A and C) displayed that wheat PLD hydrophobic surface area at 300 K was 15,721.8 Å^2^. After heating, the model structure was looser and the inner part of the hydrophobic region exposed, resulting in protein hydrophobic surface area increased to 16,300.2 Å^2^.

## Conclusions

Whole wheat flour is nutritious and includes all the nutrients of wheat kernels. But it is more prone to rancid, resulting in short shelf life and limiting its use. In this paper, the lipase in the whole wheat flour was inactivated by microwave heating, which greatly prolonged the storage time of whole wheat flour. In addition, microwave treatment can maintain the dough quality and texture quality of whole wheat flour products. These all proved that microwave heating was an effective way to prolong the storage time and assure the quality of whole wheat flour. Furthermore, the molecular docking results showed that the heating inactivation of lipase was due to the conformation changes of lipase catalytic sites. The entrance of the catalytic cavity contracted, which made the fat substrate cannot enter the active cavity easily as control, which prevented the fat hydrolysis.

## Data Availability

Not applicable.

## Abbreviations

PLD: Phospholipase D
w.b.: Wet basis
LA: Lipase
RVA: Rapid visco analyzer
TA: Texture analyzer
T: Treated
C: Control
FFA: Free fatty acid
REGWQ: Ryan-Einot-Gabriel-Welsch
RMSD: Root Mean Square Deviation
